# Epidermal growth factor receptor (EGFR) downstream signalling pathway in primary colorectal tumours and related metastatic sites: optimising EGFR-targeted treatment options

**DOI:** 10.1038/sj.bjc.6603847

**Published:** 2007-06-19

**Authors:** M Scartozzi, I Bearzi, R Berardi, A Mandolesi, C Pierantoni, S Cascinu

**Affiliations:** 1 Clinica di Oncologia Medica, Azienda Ospedaliera Ospedali Riuniti-Università Politecnica delle Marche, Via conca 60020, Ancona, Italy; 2 Istituto di Anatomia Patologica, Azienda Ospedaliera Ospedali Riuniti-Università Politecnica delle Marche, Ancona, Italy

**Keywords:** EGFR downstream signalling pathway, phosphorylated Akt, phosphorylated MAPK, anti-EGFR treatment options, colorectal tumours

## Abstract

We analysed the expression of activated (phosphorylated) Akt and MAPK in 98 cases of paired primary colorectal tumours and metastases with the aim to define better the epidermal growth factor receptor (EGFR)-related molecular profile of colorectal cancer as a tool for treatment selection. Among 47 (48%) EGFR-negative primary tumours, 35 cases (74%) were positive for phosphorylated Akt and MAPK. Among 51 (52%) EGFR-positive primary colorectal cancers, 13 (25%) cases were negative for phosphorylated Akt and 15 (29%) were negative for phosphorylated MAPK. In EGFR-negative metastases (56 cases, 55%), phosphorylated Akt was expressed in 41 (73%) and phosphorylated MAPK was expressed in 36 (64%) samples, whereas in EGFR-positive metastases, phosphorylated Akt and MAPK were negative in 14 (31%) and in 10 (22%) cases, respectively. Phosphorylated Akt expression in primary colorectal tumours changed from positive to negative in 16 (16%) paired metastases and from negative to positive in 13 (13%) related metastatic sites. Phosphorylated MAPK expression in primary tumours changed from positive to negative in 13 (13%) paired metastases and from negative to positive in 12 (12%) related metastatic sites. Our findings suggest that phosphorylated Akt and MAPK status in primary tumours does not correlate with Akt and MAPK status in corresponding metastases. EGFR downstream signalling pathway can be overactivated even in the absence of EGFR expression in a considerable proportion of patients.

The epidermal growth factor receptor (EGFR) is a tyrosine kinase protein, which plays an important role both in the signal transduction pathway and cellular function (Carpenter and Cohen, 1990).

Binding of specific ligand, such as the epidermal growth factor (EGF) and transforming growth factor *α* (TGF-*α*) to the EGFR, results in the dimerisation of the receptor with the subsequent initiation of the intracellular signalling pathways cascade. A major downstream signalling route is via the Ras-Raf-MAPK. Activation of Ras initiates a multistep phosphorylation cascade that leads to the activation of MAPKs, ERK1 and ERK2, which ultimately regulate transcription of molecules involved in cell proliferation (Mendelsohon and Baselga, 2003). Another important target in EGFR signalling is phosphatidylinositol 3-kinase (P13K) and the downstream protein-serine/threonine kinase Akt. This latter protein kinase transduces molecular signals triggering crucial steps for cell growth and survival (Carpenter and Cohen, 1990; Mendelsohon and Baselga, 2003).

The demonstration that EGFR results abnormally expressed or upregulated in 50–80% of all cases of advanced colorectal tumours (Hemming *et al*, 1992; Kluftinger *et al*, 1992; Mayer *et al*, 1993) brought to the active development of anti-EGFR treatment strategies for these patients, including monoclonal antibodies, which target the extracellular domain of the EGFR and small molecules (tyrosine kinase inhibitors, TKIs), which target the tyrosine kinase domain of the receptor. Although multiple therapeutic options targeting the EGFR molecular pathway have been proposed, the biologic mechanisms underlying the activity of these drugs ‘*in vivo*’ are still to be investigated fully.

Clinical studies demonstrated that EGFR expression is not to be considered as a predictive marker for such treatment strategies; therefore, it has been postulated that the activation of the downstream signalling pathway (Akt and MAPK) could be responsible for EGFR aberrant activity even in the absence of a detectable EGFR expression (Cunningham *et al*, 2004; Ellis and Hoff, 2004; Saltz *et al*, 2004; Fischel *et al*, 2005). In this case, targeting the receptor via monoclonal antibodies would probably be clinically irrelevant, whereas it would be more appropriate an attempt to block intracitoplasmic tyrosine kinase activity via small molecules inhibiting the tyrosine kinase portion of the EGFR. In fact, in non-small-cell lung cancer patients, it has been suggested that TKIs' responsiveness might be predicted by EGFR downstream proteins such as activated (phosphorylated) Akt (Cappuzzo *et al*, 2005; Han *et al*, 2005). However, data regarding the *in vivo* EGFR-driven molecular profile in colorectal cancer are conflicting and consequently, at present, no speculations are possible about its role in determining resistance or sensitivity to EGFR-targeted drugs.

Recently in a series of 28 advanced colorectal patients treated with gefitinib monotherapy, biologic evaluation of total and activated EGR, activated Akt, MAPK and Ki 67 on paired pre- and 1-week post-treatment tumour samples could not confirm a gefitinib-induced decreased expression of these molecular markers (Rothenberg *et al*, 2005).

However, these data does not seem to be concordant with those reported in a similar analysis by Daneshmand *et al* (2003) although in a smaller series.

After our previous finding of a substantial lack of correlation for EGFR status between primary colorectal tumours and corresponding metastases (Scartozzi *et al*, 2004), it has been suggested that the possibility for an appropriate anti-EGFR treatment selection could be highly dependent on the actual presence and activation of the target itself. We then analysed the expression of activated (phosphorylated) Akt and MAPK in primary tumours and corresponding metastatic sites with an already known EGFR status, with the aim to define better the EGFR-related molecular profile of colorectal cancer, to serve as a tool for treatment selection.

## PATIENTS AND METHODS

### Patient selection

Patients were selected from a pathological database of colorectal cancer cases, who underwent surgical resection of the primary tumour and the corresponding metastatic site, observed at the Pathology Department of the Università Politecnica delle Marche, Ancona, Italy, between 1995 and 2005.

### Immunohistochemical analysis

The expression of phospho-Akt (Ser437), p44/42 MAP kinase (Cell Signaling Technology, MA, USA) and EGFR (Dako Cytomation, CA, USA) was evaluated with an immunohistochemistry technique on 5-*μ*m-thick tissue section obtained from paraffin-embedded specimens fixed in 10% (v v^−1^) neutral buffered formalin.

The sections were deparaffinised and hydrated by passing through xylene and a graded series of ethanol, followed by washing in distilled water.

The antigens were unmasked for phospho-Akt (Ser437) by heat treatment at 98°C for 10 min, in EDTA buffer and for p44/42 MAP kinase by microwave treatment at 98°C for 10 min, in a 10 mM citrate buffer, pH 6.0. After antigens retrieval, tissues were blocked with 5% normal goat serum for 60 min.

Subsequently, the sections were incubated either with phospho-Akt (Ser437) antibody (1 : 50 dilution) or MAP kinase antibody (1 : 100 dilution) overnight at 4°C.

Consecutively, immunostaining was performed by the avidin–biotin peroxidase complex technique (Dako Envision System, CA, USA) for 30 min according to the manufacturer's instructions and using 3′,3′ diaminobenzidine (DAB, Dako Cytomation) as a chromogen. Subsequently, the slides were counterstained with Meyer's haematoxylin for 1 min, dehydrated in a graded series of alcohol, treated with xylene and cover slipped.

Positive control of phospho-Akt (Ser437) and p44/42 MAP kinase staining was performed on paraffin-embedded human breast cancer in all runs. (Data Sheet of phospho-Akt (Ser437) and p44/42 MAP kinase antibodies, Cell-Signalling Technology.)

Negative control for the validation of the phospho-Akt (Ser437), p44/42 MAP kinase assay consisted of sections incubated with secondary antibody alone without primary antibody in all runs. (Data Sheet of phospho-Akt (Ser437) and p44/42 MAP kinase antibodies; Cell Signaling Technology.)

All slides were evaluated independently by two pathologists (IB and AM).

### Evaluation of EGFR expression

Epidermal growth factor receptor expression was detected as membranous or cytoplasmic brown staining of neoplastic cells with various intensity. Positivity for EGFR expression was defined as any membrane staining above background level, whether or not completely circumferential. In each case, two stained sections of the tumours were quantified by light microscopy, and a score (range 0–100) expressing the percentage of positive neoplastic cells was obtained.

Both the primary and metastatic neoplasm were considered positive when more than 1% of the tumour cells had membranous-complete or -incomplete staining; the neoplasms that showed a specific membrane staining lower than 1% of neoplastic cells were defined negative.

The cytoplasmic staining, resulting from either internalised or nascent receptor molecules, without associated membrane staining was reported as negative.

The intensity of EGFR reactivity was scored using a three-tier system:

- 1+ weak intensity: faint brown membranous staining;
- 2+ moderate intensity: brown membranous staining of intermediate darkness producing a complete or incomplete circular outline of the neoplastic cell;
- 3+ strong intensity: dark brown or black membranous staining producing a thick outline, complete or incomplete of the neoplastic cell.

The percentage of the cells for each intensity staining (1+; 2+; 3+) was obtained when the intensity EGFR stain was heterogeneous (Scartozzi *et al*, 2004).

### Evaluation of phospho-Akt (Ser473) expression

Phospho-Akt expression was detected as cytoplasmic and nuclear staining of neoplastic cells with various intensity. The intensity of phospho-Akt (Ser473) reactivity was scored using a four-tier system: 0, no staining; 1, weak; 2, moderate; and 3, strong.

Positivity for expression of phospho-Akt (Ser473) was defined as cytoplasmatic staining, with score 2 and/or 3; negativity with score 0 and/or 1. Both the primary and metastatic neoplasm were considered positive when more than 1% of the tumour cells had score 2 and/or 3 (Roy *et al*, 2002).

### Evaluation of p44/42 MAP kinase expression

p44/42 MAP kinase expression was detected as cytoplasmic with nuclear brown staining of neoplastic cells. The intensity of p44/42 MAP kinase reactivity was scored using a four-tier system as follows: 0, no staining; 1, weak; 2, moderate and 3, strong. The proportion of neoplastic cells showing a positive signal was scored by assessing on a scale of 0–1: 0, none; 0.1, less than one-tenth; 0.5, less than one-half and 1.0, greater than one-half. The intensity and proportion scores were then multiplied to give an *H*-score; tumours with a score equal to or higher than 1.0 were deemed positive (Adeyinka *et al*, 2002; Al-haddad *et al*, 2005).

## RESULTS

Ninety-eight patients were available for our analysis: 61 (62%) men and 37 (38%) women, median age at diagnosis was 63 (range 32–86) years. Seventy-four (75%) patients had colon and 24 (25%) had rectal cancer. Colorectal adenocarcinoma was the most common histological type as it was observed in 92 (94%) pathological samples from primary tumours; the remaining six (6%) cases were mucinous adenocarcinomas. Histology grades 1–2 and 3 were described in 83 (85%) and 15 (15%) tumours, respectively. In 95 cases of primary neoplasm, only a single metastatic site (97%) was available for analysis, three cases had both pulmonary and hepatic metastases (3%) (Table 1).

Globally, pathologic samples from 101 metastatic sites were analysed: 84 (83%), liver metastases; 12 (12%), lung metastases; 4 (4%), brain and 1 (1%) bone metastases (Table 1).

All metastatic samples were obtained from metastasectomies except for the only bone metastasis, which was a biopsy. Liver metastases were synchronous in 42 cases and metachronous in the remaining 41 cases. Seven lung metastases were synchronous, whereas the remaining five were metachronous as they were all brain lesions (four cases). Therefore, globally in 49 cases (50%), the metastatic site analysed was synchronous. The median time elapsed between resection of the primary and corresponding metastatic site was 9 months.

In 38 cases (39%), surgical resection of metastases (and consequently specimens collection) was performed after the administration of chemotherapy.

Epidermal growth factor receptor expression was positive in 51 (52%) primary tumours and 45 (45%) metastatic sites (Table 2).

In primary tumours phosphorylated Akt and MAPK were positive in 73 (74%) and 71 (70%) cases, respectively, whereas phosphorylated Akt and MAPK were positive in 72 (73%) and 71 (70%) metastatic sites, respectively (Table 2).

**Figure 1:**
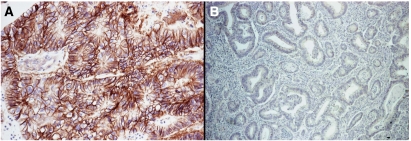
(**A**) Primary colorectal tumour showing membranous EGFR-negative staining; (**B**) primary colorectal tumour with positive cytoplasmic (arrow) and nuclear (head of the arrow) phosphorylated MAPK staining.

**Figure 2:**
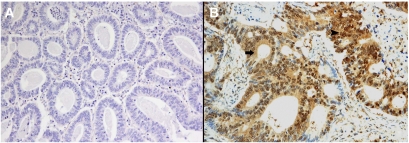
(**A**) Primary colorectal tumour showing membranous EGFR-positive staining with strong intensity (3+); (**B**) primary colorectal tumour with negative phosphorylated MAPK staining.

Among 47 (48%) EGFR-negative primary tumours, 35 cases (74%) expressed phosphorylated Akt and MAPK (Figure 1). On the contrary, among 51 (52%) EGFR-positive primary colorectal cancers, 13 (25%) cases were negative for phosphorylated Akt and 15 (29%) were negative for phosphorylated MAPK (Table 3) (Figure 2).

Similar to these findings, also in EGFR-negative metastases (56 cases, 55%), phosphorylated Akt was expressed in 41 (73%) and MAPK was expressed in 36 (64%) samples, whereas in EGFR-positive metastases phosphorylated Akt was negative in 14 (31%) cases and MAPK was negative in 10 (22%) cases (Table 4).

**Figure 3:**
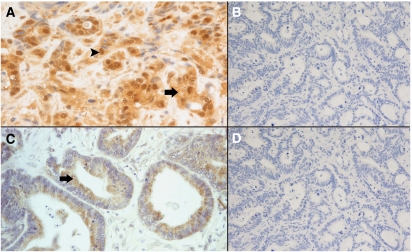
(**A**) Primary colorectal tumour showing positive cytoplasmic (arrow) and nuclear (head of the arrow) phosphorylated MAPK staining; (**B**) corresponding liver metastasis with negative phosphorylated MAPK staining. (**C**) Primary colorectal tumour showing positive cytoplasmic phosphorylated Akt staining (arrow); (**D**) corresponding liver metastasis with negative phosphorylated Akt staining.

Phosphorylated Akt expression in primary colorectal tumours changed from positive to negative in 16 (16%) paired metastases and from negative to positive in 13 (13%) related metastatic sites. Mitogen-activated protein kinase expression in primary tumours changed from positive to negative in 13 (13%) paired metastases and from negative to positive in 12 (12%) related metastatic sites (Table 5) (Figure 3). Interestingly, in all three cases with two metastatic sites available for analysis (liver and lung), we observed a variation for MAPK expression between the two different metastatic sites.

Among the group of patients receiving chemotherapy before resection of metastases, we observed a shift in phosphorylated Akt and MAPK expression in six and four cases, respectively.

## DISCUSSION

The introduction of a novel class of targeted antineoplastic agents, such as those directed against the EGFR, have notably expanded the available therapeutic options for patients with advanced colorectal cancer (Cunningham *et al*, 2004; Ellis and Hoff, 2004).

Unfortunately, available clinical data generally failed to demonstrate a correlation between the level of expression of the target (EGFR in this case) and the global outcome of patients treated, thus making the need for a reliable predictive factor for pretreatment selection of patients as potential candidates to EGFR-targeted therapies even more relevant (Cunningham *et al*, 2004; Ellis and Hoff, 2004; Saltz *et al*, 2004). Furthermore, we are unable both to select appropriately patients for an anti-EGFR treatment, and to define which treatment would be better (i.e. monoclonal antibodies or TKI) for our patient. The present analysis is based on the hypothesis that an appropriate anti-EGFR treatment selection could be dependent on the actual presence and activation of the target itself. We then analysed the expression of activated (phosphorylated) Akt and MAPK in primary tumours and the corresponding metastatic sites with an already known EGFR status, with the aim to define better the EGFR-related molecular profile of colorectal cancer, to serve as a tool for treatment selection.

One of the main results of our study was the observation that Akt and MAPK expression could be independent of EGFR status both in primary and metastatic sites, thus suggesting that EGFR downstream signalling pathway can be overactivated even in the absence of EGFR expression in a non-negligible proportion of patients. Consequently, the use of anti-EGFR treatment with monoclonal antibodies could be at least theoretically inappropriate in these tumours, whereas the use of a treatment strategy including TKIs that can interfere with the EGFR downstream pathway could be more appealing. However, these latter assumptions should be considered purely speculative, as in our analysis we did not include information about other biological determinants, such as EGFR mutations or abnormal expression of other EGFR-driven downstream molecules, which may play a relevant role in EGFR-directed treatment selection.

However, our observation seems to be similar to results reported by Rothenberg *et al* (2005), in which they were not able to confirm a correlation between the inhibition of Akt and MAPK and response to an EGFR TKI (gefitinib) in colorectal cancer, but suggested a definite trend for inhibition of the EGFR-driven activation of downstream regulators in patients achieving a longer progression-free survival. These results should be considered even more relevant if we consider that the timing adopted for tumour biopsies collection for biological studies could have been not optimal.

Similar to our previous findings of a substantial lack of correlation for EGFR status between primary colorectal tumours and corresponding metastases (Scartozzi *et al*, 2004), we also noticed a substantial variation for Akt and MAPK expression among primary tumours and related metastases.

This implies that this biological phenomenon could account for resistance to antineoplastic treatment directed against the EGFR, if we assume that the loss of the target should render ineffective any therapy directed against it. However, we should also consider that the staining methods currently used for EGFR expression could be considered inadequate as a predictive tool for anti-EGFR treatment strategies and may be primarily responsible for the apparent lack of association between EGFR positivity and response to treatment.

The observation that in 49 cases (50%), metastases were metachronous seems also to suggest the hypothesis that changes in phosphorylated Akt and MAPK expression could have occurred over time with progression of disease. Among patients receiving chemotherapy before specimens collection (38 cases, 39%), it is important to note that phosphorylated Akt and MAPK variation might be hypothetically related to a ‘selective’ effect of the treatment. Nevertheless, the number of cases observed (six cases for Akt and four cases for MAPK variation) does not seem to confirm this assumption.

As EGFR-targeted treatment strategies are employed to treat metastatic disease on the basis of our data, only the EGFR-downstream signalling pathway status in metastases would be relevant. Nevertheless, only a prospective trial including biological assessment of these parameters on metastases could definitely establish whether this could be considered effective in the clinical practice.

Taken together, we believe that our observations could bring further insights into the biology of EGFR-expressing colorectal tumours and along with growing clinical data could help clinicians in the future to select better the appropriate anti-EGFR treatment option for the appropriate patient.

## Figures and Tables

**Table 1:** Patients' characteristics

	**No. of patients (*N*=98)**	**(%)**
*Age (years)*		
Median	63	
Range	32–86	
		
*Sex*		
Male	61	62
Female	37	38
		
*Primary tumour location*		
Colon	74	75
Rectum	24	25
		
*Primary tumour characteristics*		
Adenocarcinoma	92	94
Mucinous	6	6
Gradings 1–2	83	85
Grading 3	15	15
		
*Metastatic sites analysed*	101	
Liver	84	83
Lung	12	12
Brain	4	4
Bone	1	1

**Table 2:** Global results for EGFR, Akt and MAPK expression

	**Primary tumour (%)**	**Liver metastases (%)**	**Lung metastases (%)**	**Brain metastases (%)**	**Bone metastasis (%)**
*EGFR*					
Positive	51 (52)	39 (48)	5 (42)	1 (25)	0 (0)
Negative	47 (48)	45 (52)	7 (58)	3 (75)	1 (100)
					
*Akt*					
Positive	73 (74)	61 (73)	5 (42)	4 (100)	1 (100)
Negative	25 (26)	23 (27)	7 (58)	0 (0)	0 (0)
					
*MAPK*					
Positive	71 (70)	62 (74)	8 (67)	0 (0)	1 (100)
Negative	27 (30)	22 (26)	4 (33)	4 (100)	0 (0)

**Table 3:** Akt and MAPK status according to EGFR expression in primary colorectal tumours

	**Akt**		**MAPK**	
	**Positive (%)**	**Negative (%)**	**Positive (%)**	**Negative (%)**
*EGFR*				
Positive	38 (75)	13 (25)	36 (70)	15 (30)
Negative	35 (75)	12 (25)	35 (75)	12 (25)

**Table 4:** Akt and MAPK status according to EGFR expression in metastases

	**Akt**		**MAPK**	
	**Positive (%)**	**Negative (%)**	**Positive (%)**	**Negative (%)**
*EGFR*				
Positive	31 (69)	14 (31)	35 (78)	10 (22)
Negative	41 (73)	15 (27)	36 (64)	20 (36)

**Table 5:** Akt and MAPK status variations between primary tumour and the corresponding metastatic sites

	**Akt status variation**		**MAPK status variation**	
**Metastatic sites**	**Positive to negative**	**Negative to positive**	**Positive to negative**	**Negative to positive**
Liver	12	7	9	8
Lung	4	1	2	4
Brain	0	3	2	0
Bone	0	1	0	0
Total	16 (16%)	13 (13%)	13 (13%)	12 (12%)
